# Incisor root length in individuals with and without anterior open bite: a comparative CBCT study

**DOI:** 10.1590/2177-6709.25.4.23.e1-7.onl

**Published:** 2020

**Authors:** Luis Ernesto Arriola-Guillén, Ivy Samantha Valera-Montoya, Yalil Augusto Rodríguez-Cárdenas, Gustavo Armando Ruíz-Mora, Aron Aliaga-Del Castillo, Guillerme Janson

**Affiliations:** 1 Universidad Científica del Sur, School of Dentistry, Division of Orthodontics (Lima, Peru).; 2 Universidad Científica del Sur, School of Dentistry, Division of Oral and Maxillofacial Radiology (Lima, Peru).; 3 Universidad Nacional de Colombia, Faculty of Dentistry, Division of Oral and Maxillofacial Radiology (Bogotá, Colombia).; 4 Universidad Nacional de Colombia, Faculty of Dentistry, Division of Orthodontics (Bogotá, Colombia).; 5 Universidade de São Paulo, Faculdade de Odontologia de Bauru, Departamento de Ortodontia (Bauru/SP, Brazil).

**Keywords:** Open bite, Incisor, Root length, Cone-beam computed tomography

## Abstract

**Objective:**

This study aimed to compare the root length of maxillary and mandibular incisors between individuals with open bite *versus* matched individuals with adequate overbite.

**Methods:**

This comparative, matched and retrospective study included 48 cone beam computed tomographies (CBCTs) obtained at a university radiological center. Scans belonged to 24 individuals with open bite (overbite ≤ 0 mm) and 24 individuals with adequate overbite (controls). Both groups were matched by age, sex, malocclusion classification and skeletal characteristics (ANB and FMA angles). Root length of each maxillary and mandibular incisor was measured in millimeters (mm) in a sagittal section from a perpendicular line to the enamel cement junction until the root apex (384 length measurements were made). The means of root length in both groups were compared using *t*-tests. In addition, correlations between variables were evaluated with the Pearson correlation coefficient (α = 0.05).

**Results:**

In both groups, the root length of the upper central incisors was approximately 12 mm and the root length of the maxillary lateral incisors was approximately 13 mm (*p*˃ 0.05). Likewise, the root length of lower central incisors in both groups measured approximately 12 mm (*p*˃ 0.05). However, the mandibular lateral incisor roots of open bite patients were significantly longer than in the normal overbite patients (approximately 1 mm, *p*= 0.012 right side, *p*= 0.001 left side).

**Conclusions:**

Root length of maxillary incisors and central mandibular incisor is similar in individuals with or without open bite, but the mandibular lateral incisor roots in open bite patients were significantly longer than in the normal overbite patients.

## RESUMO

**Objetivo:**

O presente estudo teve como objetivo comparar o comprimento radicular de incisivos superiores e inferiores em indivíduos com mordida aberta *versus* indivíduos com sobremordida normal.

**Método:**

Esse estudo comparativo e retrospectivo incluiu 48 tomografias computadorizadas de feixe cônico (TCFC) obtidas em um centro radiológico universitário. As tomografias foram realizadas em 24 indivíduos com mordida aberta (sobremordida ≤ 0 mm) e 24 indivíduos com sobremordida normal (controle). Ambos os grupos foram compatibilizados de acordo com a idade, sexo, má oclusão e padrão esquelético (ângulos ANB e FMA). O comprimento radicular de todos os incisivos superiores e inferiores foi medido em milímetros (mm) em um corte sagital, de uma linha perpendicular à junção cemento-esmalte até o ápice radicular (384 medidas de comprimento foram realizadas). A média de comprimento radicular em ambos os grupos foi comparada utilizando-se o teste *t*. Adicionalmente, correlações entre as variáveis foram avaliadas usando o coeficiente de correlação de Pearson (α = 0,05).

**Resultados:**

Em ambos os grupos, o comprimento radicular dos incisivos centrais superiores foi de, aproximadamente, 12 mm, e o comprimento radicular dos incisivos laterais superiores foi de, aproximadamente, 13 mm (*p*˃ 0,05). Da mesma forma, o comprimento radicular dos incisivos centrais inferiores, em ambos os grupos, obteve a medida de aproximadamente 12 mm (*p*>0,05). No entanto, o comprimento radicular dos incisivos laterais inferiores em indivíduos com mordida aberta foi significativamente maior em comparação aos pacientes com sobremordida normal (aproximadamente 1 mm, *p*=0,012 do lado direito, *p*=0,001 do lado esquerdo).

**Conclusões:**

O comprimento radicular de incisivos superiores e incisivos centrais inferiores foi similar em indivíduos com ou sem mordida aberta, mas o comprimento radicular de incisivos laterais inferiores em indivíduos com mordida aberta foi significativamente maior em comparação a pacientes com sobremordida normal.

## INTRODUCTION

It has been reported that individuals with open bite have greater incisor dentoalveolar height, compared with balanced facial pattern subjects.^1^ ^,^ ^2^ Based on the increased vertical skeletal and dentoalveolar dimensions that open bite individuals present,^1^ ^,^ ^3^ ^-^ ^7^ it could be speculated that the root lengths of anterior teeth would be greater in open bite individuals, when compared to those without open bite. 

Contrarily, some authors found shorter maxillary central incisor length in open bite patients compared to controls without open bite,^8^ or with deep bite^9^, based on lateral cephalogram evaluation. However, the root length was not directly measured.^8^ ^,^ ^9^ In addition, only two studies that evaluated dental root length in panoramic radiographs^10^ and root area in CBCT^11^ concluded that patients with open bite, especially those with a high mandibular plane angle, have shorter dental roots and smaller root areas of the maxillary incisors, when compared to individuals with normal overbite. These studies mention that their findings may be related to the loss of occlusal contact in the anterior teeth. It is important to emphasize that open bite individuals present counterclockwise rotation of the palatal plane and clockwise rotation of the mandibular plane,^5^ ^,^ ^12^ ^-^ ^14^ increasing the lack of contact between maxillary and mandibular incisors.^10^ However, a clear relationship between open bite and the presence of shorter or longer roots is not yet established, since studies that evaluate, specifically, the root length of individuals with open bite have been rarely reported. Thus, these results should be evaluated in other samples for better consistency.^15^

Therefore, the purpose of this study was to compare the root length of maxillary and mandibular incisors between individuals with open bite *versus* matched individuals with adequate overbite. 

## MATERIAL AND METHODS

This comparative and retrospective study was approved by the Ethics and Research Committee of the *Universidad Científica del Sur*, Lima/Peru (# 00021). The sample involved 48 CBCTs obtained from the files of a radiologic center at the *Universidad Científica del Sur*, of patients who underwent orthodontic-surgical treatment planning.^16^ The CBCTs were divided into two groups: Group 1, consisting of 24 scans of individuals with anterior open bite; and Group 2, consisting of 24 scans of individuals with an adequate overbite (controls). The patients were matched by age, sex, malocclusion classification and skeletal characteristics (ANB and Frankfort mandibular plane-FMA angles). 

Sample size was calculated considering an 80% of test power at a confidence level of 95%, with a mean intergroup difference to be detected of 2mm in the root length of maxillary central incisors, with a standard deviation of 1.60mm, as previously reported.^10^ Although the required sample was 10 individuals per group, 24 subjects per group were included.

The inclusion criteria of the anterior open bite group included individuals with overbite of 0mm or less (negative), mandibular plane angle defined by FMA angle ˃26° for both sexes, age range from 20 to 40 years, with all permanent teeth (excluding third molars), with Class I, II or III malocclusions. The control group included individuals with overbite from 1 to 4 mm, and with the same criteria of the open bite group. In both groups, individuals with syndromic craniofacial deformations, maxillofacial surgeries, history of previous orthodontic or orthopedic treatment, incisors with endodontic treatments, impacted canines or tooth loss prior to CBCT were excluded.

CBCT scans of all patients were taken using a tomographic equipment model Picasso Master 3D (Vatech Co., Ltd., Hwaseong, South Korea), set at 8mA, 90KVp, isotropic voxel size of 0.3mm and exposure time of 20 seconds. Each field of view mode was of 20 x 19cm. All variables were measured in the RealScan software (version 2.0, PointNix Co., Ltd., South Korea).

The overbite was measured, using the volumetric reconstruction (VR), as the distance in mm between the incisal edges of the maxillary and mandibular incisors, perpendicular to the occlusal plane. Malocclusion classification was evaluated in the dental casts.

Lateral cephalograms generated from CBCT were used to measure the cephalometric variables.^17^ Skeletal relationship was evaluated with the ANB angle and the facial pattern with the FMA angle.

**Figure 1:**
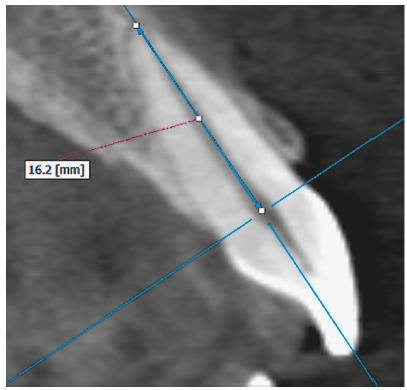
Root length measurement of maxillary incisor in the sagittal section.

**Figure 2:**
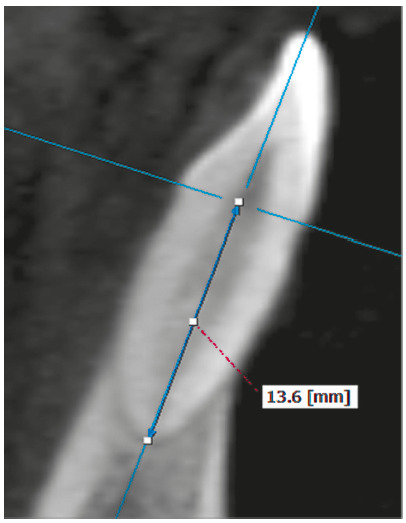
Root length measurement of mandibular incisor in the sagittal section.

The root length of each central and lateral maxillary and mandibular incisors was measured in millimeters. To obtain the tomographic cuts, the longitudinal axis of each incisor was located in the axial, sagittal and coronal views. Then, in the sagittal section the root length was measured on the same longitudinal axis, from a perpendicular projection of the labial cement-enamel junction up to the vertex of the root apex of each incisor (Figs 1 and 2).

### Error study

All measurements were made twice, at two different times, separated by a one-month interval, by two different examiners. The values obtained were evaluated through the intraclass correlation coefficient (ICC), to determine the intraexaminer and interexaminers concordance. Values greater than 0.85 (CI to 95%, 0.70-0.98) were obtained. Random errors were calculated according to Dahlberg’s formula,^18^ giving values smaller than 1mm in all quantitative variables. 

### Statistical analysis

All statistical analyses were performed using SPSS software for Windows (version 19.0; IBM, Armonk, NY). Normal distribution was tested and confirmed with the Shapiro-Wilk tests. Intergroup comparisons regarding sex and malocclusion distributions were performed with Chi-square tests. Intergroup comparisons regarding age, overbite, ANB and FMA angles and root lengths were performed with *t*-test. Finally, correlations between overbite and root lengths were evaluated with the Pearson correlation coefficient. The significance level was set at *p*< 0.05.

## RESULTS

The groups were comparable regarding sex, malocclusion classification, age, ANB and FMA angles (Tables 1 and 2). The control group presented statistically significant greater overbite than the open bite group (Table 2). 

**Table 1:** Distribution of both groups according to sex and malocclusion.

Variable	Values	Control group	Open bite group	Total	p
Sex	Male	12	12	24	1.000
	Female	12	12	24	
Angle malocclusion	Class I	6	6	12	1.000
	Class II	8	8	16	
	Class III	10	10	20	

Chi-square test.

**Table 2:** Group comparability regarding the initial characteristics and intergroup comparisons of root lengths.

Measurements	Control group (n=24)		Open bite group (n=24)		Mean difference	Lower Limit CI to 95%	Upper Limit CI to 95%	p
	Mean	SD	Mean	SD				
Initial characteristics								
Age	33.80	9.07	30.89	7.40	2.91	-3.24	8.57	0.343
Overbite	2.71	1.49	-2.65	2.26	5.36	4.13	6.58	<0.001*
ANB Angle								
Class I	1.31	0.21	1.23	0.77	0.08	-2.35	2.50	0.907
Class II	6.20	1.22	6.42	1.49	-0.22	-1.34	1.45	0.857
Class III	-3.37	2.02	-2.35	1.85	-1.01	-2.84	0.80	0.257
FMA	28.10	2.43	30.15	4.34	-2.05	-4.35	2.50	0.079
Root lengths								
Maxillary right central incisor	12.94	1.24	12.29	1.72	0.65	-0.30	1.61	0.178
Maxillary left central incisor	12.81	0.98	12.50	2.26	0.31	-0.81	1.42	0.583
Maxillary right lateral incisor	13.06	1.31	12.96	1.79	0.10	-0.91	1.10	0.849
Maxillary left lateral incisor	13.12	0.80	13.20	1.65	-0.08	-0.90	0.75	0.846
Mandibular right central incisor	11.71	0.62	11.71	1.43	0.00	-0.70	0.70	1.000
Mandibular left central incisor	11.82	0.75	11.49	1.38	0.33	-0.38	1.04	0.353
Mandibular right lateral incisor	11.79	0.77	12.87	1.66	-1.08	-1.92	-0.25	0.012*
Mandibular left lateral incisor	11.70	0.98	12.07	1.41	-0.37	-2.16	-0.59	0.001*

*Statistically significant at p < 0.05 (t-test).

Root lengths ranged from 12.29 mm to 13.20 mm for the maxillary incisors, and did not show significant intergroup differences (Table 2).

For the mandibular central incisors, the root lengths ranged from 11.49 mm to 11.71 mm, and only the root lengths of the open bite mandibular lateral incisors were significantly greater than the normal overbite group.

There were significant inverse correlations between overbite and the root lengths of the mandibular lateral incisors, but with low to moderate strengths (Table 3).

**Table 3:** Correlation values between the overbite and the root length of maxillary (Mx.) and mandibular (Md.) incisors.

Pearson correlation		Mx. right central incisor	Mx. left central incisor	Mx. right lateral incisor	Mx. left lateral incisor	Md. right central incisor	Md. left central incisor	Md. right lateral incisor	Md. left lateral incisor
Overbite	R	0.278	0.260	0.010	-0.140	-0.069	-0.048	-0.345	-0.490
	P	0.176	0.105	0.949	0.390	0.671	0.771	0.029*	0.001*

*Statistically significant at p < 0.05.

## DISCUSSION

A perfect similarity of the biological and physical characteristics of the individuals in both groups was difficult to achieve due to the great individual variability of the participants. Nevertheless, this is one of the few studies that directly evaluate root lengths in subjects with and without open bite using CBCT scans.

Some authors compared the dentoalveolar height of incisors with respect to the palatal plane, between subjects with and without open bite, finding that individuals with open bite have greater dentoalveolar height of incisors.^1^ ^,^ ^2^ However, these results only identify that the incisors in open bite subjects have greater dentoalveolar height, but they did not evaluate their root lengths. In this way, Harries and Butler^9^ found, on lateral radiographs, that the length of permanent maxillary central incisors was significantly shorter in adolescents with open bite than matched adolescents with deep bite before orthodontic treatment.

There are a few investigations that have compared the incisor or root lengths between individuals with and without open bite. A first study was carried out by Arntsen et al.^8^ on lateral radiographs and evaluated the entire incisor length, including the crown and the root. They concluded that the length of the upper incisors was smaller in open bite individuals when compared to controls without open bite. Based on these results, it could be thought that if the maxillary incisor length is shorter in open bite individuals, the same may be expected for the root size. However, this is a speculation. In addition, lateral radiographs have the disadvantage of presenting image superimposition of both central incisors, thus the length evaluation of any incisor requires a very good calibration. 

Subsequently, Uehara et al.^10^ through panoramic radiographs, compared the root-crown ratio and root length between individuals with open bite and controls with normal overbite. They found that open bite individuals had smaller crown-root ratio and root length from the incisors to premolars in maxillary and mandibular teeth, when compared to individuals with normal overbite. They attributed this characteristic to the loss of occlusal contact, arguing that in the lack of occlusal contact or hypofunction, there could be some atrophic changes in the periodontal ligament that could influence root length. They stated the limitations of using panoramic radiographs and suggested further research using CBCT. 

A recent study using CBCT reported that root surface areas of maxillary incisors are smaller in open bite individuals, when compared to controls without open bite.^11^ They attributed their results to the occlusal hypofunction mentioned above, and speculated that some abnormal pressure from a tongue thrusting habit could cause root resorption of these teeth. Nevertheless, their sample size and age range were smaller than in the present study, and it may have influenced their results. In addition, it should be considered that length and area measurements are different. One might find smaller area in a narrow and longer root or a greater area in a wide and shorter root. Thus, area and length measurements should be independently and carefully assessed.

Contrary to the findings of these studies, it could be thought that if open bite patients present greater vertical dimensions and dentoalveolar heights than subjects with normal overbite,^1^ ^,^ ^3^ ^-^ ^6^ the presence of similar or even greater dental tooth size and consequently greater root length could be expected. However, the results of this study showed no significant difference in root length of maxillary incisors between subjects with and without open bite (Table 2). This may be explained because the groups did not show significant difference regarding the vertical skeletal pattern. In groups with significant vertical skeletal differences, this scenario may change, and this should be evaluated in future research.

Since no significant differences were found for the maxillary incisors, the same results would be expected for the mandibular incisors. However, significant differences were found in the lateral incisors, showing that individuals with open bite have greater root length, ranging from 0.37mm to 1mm, approximately, when compared to the control individuals (Table 2). In addition, significant inverse correlations were found between root length of mandibular lateral incisors and overbite; however, they presented low to moderate strength, which is not clinically relevant (Table 3). Although these results are in accordance with the speculations of greater root length in open bite subjects, these differences lack clinical relevance. Again, further studies comparing extreme vertical malocclusions should be performed to confirm these results.

If incisors with short roots are a typical characteristic of individuals with open bite malocclusion, this should be a common finding in the different published studies involving different samples. However, the lack of articles that evaluate this association, i.e., lack of consistency (previous articles supporting this relationship),^15^ beyond those mentioned above, make it difficult to justify this conclusion. Therefore, future studies are necessary to clarify this causality relationship. Furthermore, for the existence of a cause-effect relationship between two variables (i.e., the existence of short roots and the presence of an open bite), certain specific characteristics should be necessary to eliminate any type of coincidence. Thus, the concept of temporality (firstly, existence of the independent variable; and secondly, presence of the outcome variable) is essential, but this could only be evaluated and demonstrated through follow-up studies ensuring the absence of the outcome variable at the beginning of the study. Plausibility (biological explanation of this relationship) is another concept that should be clear to ensure this relationship, that is defined as the biological explanation why individuals with an open bite could have short roots. Likewise, the strength of association, the biological gradient and coherence are other factors that a causal relationship should also fulfill.^15^ The present study, by its own design, did not seek to evaluate a true causality relationship, but sought to determine whether root length presents significant differences between comparable individuals with and without open bite, information that could be applied in clinical practice.

Consequently, associating the present results with the controversy about greater root resorption after orthodontic treatment in open bite patients,^19^ ^-^ ^21^ the orthodontist could understand that treatment planning in individuals with and without open bite should have similar considerations regarding the initial condition of root length. In both cases, factors that could cause moderate root resorption of incisors should be similarly avoided.

## CONCLUSIONS

Root length of maxillary incisors and mandibular central incisors is similar in individuals with or without open bite, but root lengths of mandibular lateral incisors in the open bite group were significantly greater than in the normal overbite group.
