# ERRATUM

**DOI:** 10.1590/2177-6709.25.6.err.001

**Published:** 2020

The original article *“Incisor root length in individuals with and without anterior open bite: a comparative CBCT study”*, with DOI: 10.1590/2177-6709.25.4.23.e1-7.onl, published in Dental Press J Orthod, vol. 25, no. 4, July/Aug. 2020, Epub Sep 21, 2020, presented the following authors: 

Luis Ernesto Arriola-Guillén, Ivy Samantha Valera-Montoya, Yalil Augusto Rodríguez-Cárdenas, Gustavo Armando Ruíz-Mora, Aron Aliaga-Del Castillo and **Guillerme** Janson

The **“How to cite this article”** presented the following information (currently in PubMed): 

Arriola-Guillén LE, Valera-Montoya IS, Rodríguez-Cárdenas YA, Ruíz-Mora GA, **Castillo AA**, Janson G. Incisor root length in individuals with and without anterior open bite: a comparative CBCT study. Dental Press J Orthod. 2020 Jul-Aug;25(4):23.e1-7. doi: 10.1590/2177-6709.25.4.23.e1-7.**onl**. PMID: 32965383.

Now the article should have the following row of authors and **“How to cite this article”**: 

Luis Ernesto Arriola-Guillén, Ivy Samantha Valera-Montoya, Yalil Augusto Rodríguez-Cárdenas, Gustavo Armando Ruíz-Mora, Aron Aliaga-Del Castillo and **Guilherme** Janson

Arriola-Guillén LE, Valera-Montoya IS, Rodríguez-Cárdenas YA, Ruíz-Mora GA, **Aliaga-Del Castillo** A, Janson G. Incisor root length in individuals with and without anterior open bite: a comparative CBCT study. Dental Press J Orthod. 2020 Jul-Aug;25(4):23.e1-7. doi: 10.1590/2177-6709.25.4.23.e1-7.**err**. PMID: 32965383.

